# Impact of climate‐induced compositional shifts of ethanol and acidity on aroma release and perception in red wine: a case study on γ‐nonalactone and massoia lactone

**DOI:** 10.1002/jsfa.70876

**Published:** 2026-07-06

**Authors:** Roberto Salvatore Di Fede, Elisabetta Pittari, Luigi Moio, Paola Piombino

**Affiliations:** ^1^ Department of Agricultural Sciences, Division of Vine and Wine Sciences University of Napoli Federico II Avellino Italy

**Keywords:** climate change, ethanol, matrix interactions, pH, total acidity, VOCs

## Abstract

**BACKGROUND:**

Climate change‐driven warming is increasing wine ethanol, promoting an atypical convergence between pH and titratable acidity (TA), and favouring the accumulation of specific wine volatiles: γ‐nonalactone (γ‐C9) and massoia lactone (MLac). This study evaluated how ethanol and acidity modulate release and sensory impact of γ‐C9 and MLac in red wine. A deodourised commercial red wine was reconstituted into nine model matrices using a 3 × 3 factorial design (12%/14%/16% *v/v* ethanol; pH/TA ratios 0.40/0.55/0.80) and spiked with γ‐C9 and MLac. Headspace release was quantified by solid‐phase micro‐extraction‐gas chromatography–mass spectrometry (HS‐SPME‐GC–MS), and trained assessors performed triangle tests and rate‐all‐that‐apply (RATA) profiling (orthonasal, retronasal, taste/mouthfeel).

**RESULTS:**

Orthonasal perception results showed that γ‐C9 was discriminated in all low pH/TA matrices, and also MLac was perceivable in high ethanol/low pH/TA matrices. These sensory results were explained by a higher release of the lactones in the headspace of wines with 16% *v/v* and low pH/TA; however, some sensory differences were not explained by headspace changes, suggesting matrix‐dependent perceptual interactions. RATA indicated a shift from citrus/tropical notes towards sweeter/ethereal nuances with greater body as ethanol increased and/or pH/TA rose, while taste ratings showed reduced sourness and increased bitterness at high ethanol/high pH/TA.

**CONCLUSIONS:**

Overall, these results suggest that ethanol and acidity act as interactive modulators, influencing volatile organic compounds (VOCs) release, their perceptual integration, and taste balance. In this context, enological practices such as wine acidification, essential to counteract climate‐induced imbalances, should be carefully evaluated for their potential ability to enhance the sensory impact of γ‐C9 and MLac and overall sensory quality in red wines. © 2026 The Author(s). *Journal of the Science of Food and Agriculture* published by John Wiley & Sons Ltd on behalf of Society of Chemical Industry.

## INTRODUCTION

Climate change (CC) is nowadays recognised as a major challenge for viticulture and oenology,^1^, ^2^ with rising temperatures representing one of its most impactful drivers, leading to notable alterations in grape composition and, consequently, wine sensory characteristics.^2^, ^3^, ^4^, ^5^ In addition to affecting the chemical composition of grapes and wine from a qualitative perspective, on a global scale, the increasing frequency of extreme and unpredictable weather events linked to CC has driven world wine production in 2024 to its lowest level in over 60 years.^6^

Wine is a complex alcoholic beverage mainly constituted of a volatile fraction, comprising ethanol and volatile organic compounds (VOCs) defining wine aroma, and a non‐volatile fraction, whose key constituents include polyphenols, defining colour and in‐mouth sensations, and organic acids, responsible for sourness and defining wine pH.^7^ These fractions interact through chemical and perceptual interactions shaping the chemical and sensory quality of wine.^8^, ^9^, ^10^, ^11^, ^12^, ^13^

Within this complex framework of wine matrix chemical composition, ethanol content, pH, and titratable acidity (TA) have emerged as critical parameters, mostly given their sensitivity to CC‐related rising temperatures, with notable implications for wine composition, technical performance, sensory quality, and longevity. Rising temperatures have resulted in increased grape sugar levels, leading to wines with higher ethanol concentrations.^5^ Simultaneously, high temperatures exert a substantial impact on the acid profile of grapes and wines, generally reducing TA and increasing pH with a trend towards their abnormal convergence.^5^, ^14^, ^15^ This is partly due to the fact that while tartaric acid remains relatively constant during ripening,^16^ malic acid is thermally degraded through respiration and enzymatic catabolism under high temperature conditions.^17^ These compositional changes, mainly driven by warmer temperatures often require agronomic practices and oenological interventions to manage alcohol concentration in wine^3^, ^18^ and to achieve the appropriate pH/TA ratio, needed for microbial, colour and chemical stability as well as for sensory balance,^19^, ^20^ also in response to consumer trends currently favouring lighter wines in terms of alcohol content.^19^ In conjunction with rising temperatures, water stress events tend to concentrate sugars and modify berry physiology, generally promoting an increase in potential ethanol content and further alterations in acid balance, mediated in part by potassium accumulation^2^, ^21^; exposure to light and ultraviolet (UV) radiation regulates secondary metabolic pathways of the grapes and can potentially modify the volatile profile of the resulting wines.^22^, ^23^

Regarding the volatile fraction of wine, studies have shown that high temperatures can lead to the accumulation of specific molecular markers, among which γ‐nonalactone (γ‐C9) and 5,6‐dihydro‐6‐pentyl‐2*H*‐pyran‐2‐one, known as massoia lactone (MLac). This accumulation leads to the development of prune and dried fig odours,^24^, ^25^ often considered undesirable as they are usually associated with premature ageing olfactory notes.^24^

Furthermore, γ‐C9 is already present in grape musts, and its concentration increases in wine, as *Saccharomyces cerevisiae* transforms 4‐oxononanoic acid (ranging from trace to ~60 μg L^−1^) into γ‐C9 with approximately 95% efficiency during alcoholic fermentation^26^; linoleic acid and 13‐hydroperoxyoctadecadienoic acid have also been identified as precursors of γ‐C9 during alcoholic fermentation.^27^ The γ‐C9 levels increase with grape ripeness: analyses of Pinot noir grapes over multiple vintages revealed increasing γ‐C9 concentrations with advancing maturity.^28^ Harvest timing further contributes to its variability: dry wines contain lower concentrations of γ‐C9 (2–20 μg L^−1^), compared to icewines from late harvested grapes (61–179 μg L^−1^).^29^, ^30^ Further research has shown that the γ‐C9 concentration is positively influenced by grape shrivelling, leading to elevated levels of the compound.^31^, ^32^ Additionally, oxidative processes appear to enhance γ‐C9 formation also in wine.^33^ The sensory relevance of γ‐C9 is underscored by its low odour threshold of 27 μg L^−1^,^34^ with concentrations in wines ranging from 5.9 μg L^−1^ (white wines) up to 178.5 μg L^−1^ (sweet or old red wines) depending on wine style.^29^, ^35^, ^36^, ^37^

MLac (5,6‐dihydro‐6‐pentyl‐2*H*‐pyran‐2‐one) can be found in must and wine, and its final concentration can be affected by harvest date^38^ and technological processes such as grape dehydration.^32^ Although its origin in grapes is not yet fully elucidated, it is suspected to derive from the degradation of ricinoleic and linoleic acids.^25^, ^39^ MLac has also been detected within the hydrolysates of grape‐extracted phenolic and aromatic fractions in a study aimed to assess grape aroma potential based on the accelerated hydrolysis.^40^ From a sensory perspective, its threshold is 10–11 μg L^−1^ in must and model wine, respectively, and its (*R*)‐ enantiomer has been shown to contribute to dried fig flavour in Merlot and Cabernet Sauvignon musts, reaching concentrations up to 68 μg L^−1^. However, in wines with similar flavours, the concentration is reduced to around 20 μg L^−1^ due to partial conversion to (*R*)‐δ‐decalactone during alcoholic fermentation.^15^

Several studies have highlighted that the sensory quality of wine depends not only on its chemical composition in terms of sensory active compounds, but also on the interactions between the volatile fraction and the non‐volatile matrix, including perceptual interactions. For example, ethanol concentration influences the solubility of VOCs and their partitioning between gas and liquid phases, and high ethanol levels are generally associated with a reduced release of several VOCs.^41^, ^42^, ^43^ In red wines with high ethanol content, the volatility of compounds with fruity or floral scent has been suppressed, reducing their perception.^44^, ^45^ Interestingly, even small variations in ethanol concentration (1% *v/v*) can significantly affect the release of VOCs in the wine matrix, altering the aromatic profile of the wine.^12^ Furthermore, changes in acidity can influence the release of specific volatile compounds; although the mechanisms behind these interactions are not yet fully understood, recent studies indicate that variations in pH and TA can influence volatile release by affecting ionisation and solvation dynamics within the matrix.^46^, ^47^

Although numerous studies have investigated how the main wine matrix components such as ethanol, organic acids, sugars, and polyphenols interact with VOCs,^7^, ^8^, ^9^, ^12^, ^48^, ^49^, ^50^ to the best of our knowledge, no research has yet explored how these components – altered by the effects of CC – may interact collectively to modulate aroma release and flavour perception in red wines. It is not yet clear whether higher ethanol levels might amplify, suppress, or alter the release and sensory impact of CC volatile molecular markers, and whether shifts in pH/TA levels could modulate their volatility and perception. Given the central role of the sensory quality in such hedonic beverages as wine, integrated studies addressing the combined impact of these compositional changes across different climatic scenarios are needed. Therefore, this study investigated the holistic impact of compositional parameters influenced by rising temperature associated with CC – namely γ‐C9, MLac, ethanol, pH and total acidity – on the sensory quality of red wine, examining the main sensory dimensions (orthonasal olfaction, taste, mouthfeel and retronasal olfaction) through three experimental steps. The working hypothesis was that the climate‐related increase in ethanol content, combined with the modification of the pH/TA ratio, would not act independently, but would interactively modulate the release and sensory perception of climate‐related volatile markers (γ‐C9 and MLac) in red wine.

## MATERIAL AND METHODS

### Chemicals

Chemicals used for the reconstitution of deodourised wines and/or the panel training (food grade): tartaric acid, tannic acid, caffeine, sodium chloride, sucrose, citric acid, malic acid were purchased from ACEF (Piacenza, Italy); monosodium glutamate was purchased online from Ajinomoto (Tokyo, Japan); ethanol 96% was from VWR International (Rosny‐sous‐Bois, France); γ‐C9 (≥ 98%) and MLac (≥ 95%) came from Sigma‐Aldrich (St Louis, MO, USA).

For the gas chromatography–mass spectrometry (GC–MS) analysis, 2‐octanol (≥ 97%) (Sigma‐Aldrich) was used as internal standard.

### Oenological chemical analyses

The determinations of pH, and TA were carried out following the guidelines of the OIV Compendium of International Methods of Wine and Must Analysis.^51^

### Samples

A commercial table red wine (Tavernello, Caviro, Faenza, Italy, vintage 2023) was used for the study. It was deodourised, reconstituted, and subsequently spiked with γ‐C9 and/or MLac to generate nine experimental wine matrices, each characterised by distinct ethanol levels and pH/TA ratios. The matrices were designed to simulate wines representing different CC scenarios that were analysed as described later.

#### Deodourisation of wine

The commercial table red wine (Tavernello), with pH 3.6, TA = 5.0 g L^−1^ (tartaric acid), and ethanol = 11.5% *v/v*, was deodourised following the same procedure described by Pittari *et al*.^9^ with some modifications. Briefly, 1‐L aliquots of the wine were each subjected to rotary evaporation (Rotavapor R‐210; Büchi, Flawil, Switzerland) at 32 °C under reduced pressure until approximately 95% of the initial weight was lost, yielding individual non‐volatile concentrates. These concentrates were then weighed, reconstituted with a small, fixed volume of deionised water, pooled, and thoroughly mixed to obtain a single homogeneous base, which was divided into nine aliquots, bottled, and stored at −20 °C. Once thawed, the aliquots were reconstituted to obtain the nine model matrices by adding deionised water and ethanol to reach the desired alcohol content (% *v/v*), followed by correction of pH and TA to the target values reported later (see Climate change model wine matrices section) and in Table 1.

**Table 1:** Chemical parameters of the nine model wine matrices reconstituted from a deodourised wine to represent different climate change (CC) scenarios

Code	Wine quality	Ethanol			Acid profile				
		Percentage *v/v*	Level	Scenario	pH	Total acidity (g L^−1^)	pH/titratable acidity (pH/TA) ratio	Ratio level	Scenario
LL	Proper	12	Low	Proper	3.2	8	0.4	Low	Proper
ML	Alarm	14	Medium	CC trend	3.2	8	0.4	Low	Corrected
HL	Alarm	16	High	CC	3.2	8	0.4	Low	Corrected
LM	Alarm	12	Low	Proper	3.6	6.5	0.55	Medium	CC trend
MM	Alarm	14	Medium	CC trend	3.6	6.5	0.55	Medium	CC trend
HM	Alarm	16	High	CC	3.6	6.5	0.55	Medium	CC trend
LH	Alarm	12	Low	Proper	4	5	0.8	High	CC
MH	Alarm	14	Medium	CC trend	4	5	0.8	High	CC
HH	Dangerous	16	High	CC	4	5	0.8	High	CC

As a preliminary step, the efficacy of the deodourisation process in terms of odour removal was evaluated through a triangle test (TT, ISO 4120:2021)^52^ by 24 participants (11 males and 13 females, aged 22–50 years). The wine used in the study (Tavernello) and different commercial red wines from other grape varieties (Aglianico, Primitivo, and Pinot noir), selected for their different aroma profiles,^53^, ^54^ each deodourised and reconstituted to the same ethanol content (12% *v/v*), were compared in terms of odour characteristics. No significant differences (*P* < 0.05) in olfactory perception were found between the matrices, demonstrating that the deodourisation was sufficiently effective in removing volatiles and making the reconstituted wines indistinguishable.

#### Climate change model wine matrices

In order to reproduce different scenarios related to CC, nine model wine matrices were reconstituted from the deodourised red wine according to a full factorial design based on three ethanol levels (Low = L: 12% *v/v*; Medium = M: 14% *v/v*; High = H: 16% *v/v*) and three pH/TA ratios (Low = L: 3.2/8 = 0.4; Medium = M: 3.6/6.5 = 0.55; High = H: 4/5 = 0.8), as reported in Table 1. Each matrix was assigned a two‐letter code, in which the first letter refers to ethanol and the second to the pH/TA ratio, as follows: LL (12%, 0.4), ML (14%, 0.4), HL (16%, 0.4), LM (12%, 0.55), MM (14%, 0.55), HM (16%, 0.55), LH (12%, 0.8), MH (14%, 0.8), and HH (16%, 0.8). The samples were reconstituted by adding deionised water and ethanol at an appropriate concentration to reach the desired alcohol content (% *v/v*). TA was measured by titration, while pH was measured using a pH meter (Edge, Hanna Instruments, Woonsocket, RI, USA) and both were corrected to the levels shown in Table 1 by addition of tartaric acid and a solution of sodium hydroxide (NaOH, 10 mol L^−1^), respectively. Tartaric acid was used since it is the strongest of the main wine organic acids and the most used acidifying agent for small acidity corrections in wine,^20^ therefore reflecting the acidification practices commonly adopted in winery conditions.

The combinations simulated progressive CC impact from ‘proper’, passing through ‘alarm’, and then to ‘dangerous’ scenarios^1^, ^55^, ^56^ of wine quality, defined on the basis of documented trends^1^ in grape and wine composition under warming conditions, including increases in alcohol content (from ~12–14% *v/v* to > 14% *v/v* under CC scenarios) and pH (from ~3.4–3.6 to ~3.7–3.9), accompanied by a progressive decline in acidity (Table 1).

Subsequently, each sample was spiked with 155 μg L^−1^ of γ‐C9 and 26.8 μg L^−1^ of MLac. These concentrations were chosen according to the highest values reported in the literature for red wines.^38^, ^54^ The wine matrices obtained were then stored at 12 ± 2 °C until analysis.

### Sensory analysis

Discriminant and descriptive sensory tests were applied. The TT^52^ was used to determine whether there were significant differences between each wine matrix spiked or not with γ‐C9 or MLac. The descriptive analysis was conducted using a rate‐all‐that‐apply (RATA) test, designed to generate sensory profiles for the nine matrices representative of CC scenarios, thus testing the sensory impact of the compositional variables of interest.

All sessions were performed at controlled room temperature (20 ± 2 °C), in individual booths, using covered black ISO glasses^57^ containing 40 mL of sample. The glasses were coded with three‐digit random numbers.

#### Panel

The sensory panel comprised 35 individuals with previous experience in discriminant sensory tests (20 males and 15 females, aged 22–54 years), and a subset of 16 participants (nine males and seven females, aged 22–54 years) was trained for the descriptive sensory assessment. All panelists were recruited among students and researchers from the Department of Agricultural Sciences, Division of Vine and Wine Sciences, at the University of Naples Federico II. Selection criteria included interest, availability, and the ability to recognise olfactory and oral stimuli. All participants had previous long experience in sensory wine tests. All procedures were conducted in accordance with the ethical standards of the institutional and/or national research committee and with the 1964 Helsinki Declaration and its later amendments or comparable ethical standards. The study was approved by the Ethics Committee for Nonbiomedical Human Research (CERSUB) of the University of Naples Federico II (PG/2024/0037120 dated 27 March 2024). Participation was on a voluntary basis, and prior to the experiments, tasters were required to sign an informed consent form disclosing the type of research, voluntary participation, and agreement to taste and spit reference solutions and wines. Participants were also informed of their right to withdraw from the study at any time without providing any justification. All data were collected anonymously.

#### Sensory training

The 16 judges who participated in the RATA test were trained following the guidelines outlined by OIV^58^ for 16 one‐hour sessions. During these training sessions, judges completed tasks related to taste and mouthfeel sensations, including description/recognition and ranking using stimuli dissolved in both water and table wine.^58^ Concerning odours, training was performed as previously described.^9^, ^59^ Briefly, panelists were trained to identify 62 real odour standards derived from established literature^9^, ^59^ and non‐scientific sources (disciplinaries, consortium/producers' websites, and informative journals) to capture the descriptors most used for defining the aromatic profiles of red wines. The standards represented 19 wine odour families: white and yellow fruit, red fruit (berries), citrus, exotic fruit, dehydrated fruit, dried fruit, cooked or candied fruit, floral, vegetal, balsamic, herbal tea/infusion odours, aromatic herbs, spices, toasted/woody, sweet odours, undergrowth, chemical, lactic, off‐odours. During these training sessions, panelists were presented with 13–17 odour standards per session, encompassing two to five odour families. The standards were served in covered 80 mL disposable plastic cups, and panelists were tasked with recognising the corresponding odour families or specific descriptors. Group discussions at the end of each session were conducted to avoid redundancy and clarify overlapping terms.

The training phase also included familiarisation with the RATA methodology and the use of a seven‐point numerical intensity scale ranging from one (very low) to seven (very high).

#### Sensory evaluation

##### Triangle tests (TTs)

TTs were performed on the nine wine matrices reported in Table 1. For each TT, three coded samples were presented in random order. Judges were tasked with identifying the sample they perceived as different and providing a forced response. The panel tested the nine matrices spiked with γ‐C9 and those spiked with MLac during different sessions.

##### Descriptive analysis (RATA)

The RATA method was also carried out on reconstituted matrices representing the nine CC scenarios. These matrices were spiked with both CC molecular markers under investigation. The replicates of descriptive evaluation were carried out across different measurement sessions. During the RATA test, panelists completed three different tasks: (a) orthonasal assessment: smelling each sample and rating the intensity of those terms that applied to the sample choosing them from the list used in the training phase, using a seven‐point scale in accordance with RATA methodology^60^; (b) retronasal assessment: having a sip (no swallowing) of wine and rating those terms that applied for the aroma of each sample in the same way as for the odours; (c) taste and mouthfeel assessment: taking a sip (no swallowing) while wearing nose clips and rating only terms related to taste.

### Analysis of γ‐C9 and MLac release

#### Headspace SPME isolation of γ‐C9 and MLac

The two VOCs, γ‐C9 and MLac, were isolated through a static headspace‐solid‐phase micro‐extraction (HS‐SPME) using the same method applied by Piombino *et al*.^12^ with some modifications. Briefly, for each analysis, 5 mL of the sample were prepared with the addition of 1 μL of 2‐octanol (200 mg L^−1^ in ethanol) used as an internal standard. The samples were placed in 20 mL screw‐cap clear glass vials sealed with polytetrafluoroethylene (PTFE)/silicone septa. The vial containing the sample was placed in a thermostatic bath at a constant temperature of 40 ± 2 °C for 10 min. A DVB/CAR/PDMS (divinylbenzene/carboxen/polydimethylsiloxane) fibre (50/30 μm coating thickness, 2 cm length) was then exposed to the sample headspace for 30 min using a manual SPME fibre holder (Supelco, Bellefonte, PA, USA). All pre‐concentrations were performed in triplicate. A blank run of the fibre was performed between two consecutive analyses to ensure the absence of residual compounds on the fibre.

#### Quantitative GC–MS analysis of γ‐C9 and MLac

Quantification was performed using a GC–MS‐QP2010 quadrupole mass spectrometer (Shimadzu, Kyoto, Japan) equipped with a DB‐WAX UI column (60 m, 0.25 mm inner diameter, 0.25 μm film thickness; J&W Scientific Inc., Folsom, CA, USA). The injector and the electron source were kept at temperatures of 250 and 200 °C, respectively. The SPME fibre was desorbed in the injector for 10 min, in splitless mode. The oven temperature was maintained at 40 °C for 5 min and then increased by 4 °C min^−1^ up to 220 °C and held for 7 min. Helium was used as a carrier gas with a constant flow of 1.02 mL min^−1^. Electron impact mass spectra were recorded with an ion source energy of 70 eV.^61^ The analyses were performed in SIM mode considering quantitation ions *m/z* 85 and 97, respectively, for γ‐C9 and MLac and as qualifying ions *m/z* 41, 56 for γ‐C9 and *m/z* 68 for MLac.^15^, ^62^, ^63^

To quantify the release of γ‐C9 and MLac in the nine reconstituted matrices, calibration curves were prepared for both target compounds. Six concentration levels were prepared. The wine matrix used for the calibration levels was obtained from a deodourised wine and reconstituted to the following parameters: 14% (*v/v*) ethanol, 6.5 g L^−1^ of tartaric acid and adjusted to pH 3.6. An aliquot of 1 μL of 2‐octanol (200 mg L^−1^ in ethanol) was added to each calibration solution as an internal standard. The target compounds were added at the following concentrations: 0, 37.5, 75, 150, 300 and 600 μg L^−1^ for γ‐C9, and 0, 7.5, 15, 30, 60 and 120 μg L^−1^ for MLac. Calibration curves were constructed by linear regression of the analyte‐to‐internal standard peak area ratios *versus* concentration, and the regression line was forced through the origin.

The data were processed using the GC–MS software solution program (version 2.30; Shimadzu).

### Data analysis

Data from TTs were analysed according to ISO 4120:2021.^52^ Chemical and sensory data were treated by analysis of variance (ANOVA, Tukey honestly significant difference (HSD) *post hoc* test, *P* < 0.05) to determine significant differences. Principal component analysis (PCA, Pearson, *P* < 0.05) was performed using significant sensory descriptors as variables and wine matrices as observations.

Data were processed by XLStat (version 2020.5.1), an add‐in software package for Microsoft EXCEL.

## RESULTS AND DISCUSSION

### Influence of the matrix composition on the orthonasal perception and release of γ‐C9 and MLac

The first objective of this study was to investigate how wine matrix composition, and specifically variations in ethanol content and acidic profile (pH/TA ratio), could affect the orthonasal perception of γ‐C9 and MLac, two aroma markers associated with elevated temperatures driven by CC.^15^, ^23^

To achieve this, TTs were performed on the nine model red wine matrices reproducing the different CC compositional scenarios. As shown in Table 2, variations in ethanol concentration and/or acidic profile can significantly influence the olfactory perception of the two VOCs, indicating a modulatory effect of these matrix components on their sensory impact. The presence of γ‐C9 was significantly discriminated in all samples with the lowest pH/TA ratio (0.4), independently of alcohol concentration (matrices LL, ML, HL). Even MLac was significantly discriminated in the wine with the highest alcohol level (16%) and the lowest pH/TA ratio (0.4) (HL matrix), and even more so in the wine with medium alcohol content (14%) and high pH/TA ratio (0.8) (MH matrix). Thus, both γ‐C9 and MLac were clearly perceivable when added to wine matrices with the highest ethanol content (16%) and low pH/TA ratio (3.2/8) (HL matrix). The latter is representative of a matrix in which the hazardous effects currently observed on wine composition due to global warming – such as the convergence of pH and total acidity – have been corrected, whereas the concomitant high alcohol content persists.^55^, ^56^

**Table 2:** Results of the triangle test (TT) applied to evaluate odour discriminability between the nine wine matrices added or not with γ‐nonalactone (γ‐C9) or massoia lactone (MLac)

Code	Ethanol (% *v/v*)	pH/titratable acidity (pH/TA) ratio	γ‐C9 (155 μg L^−1^)	MLac (26.8 μg L^−1^)
LL	12	0.40	*	ns
ML	14	0.40	*	ns
HL	16	0.40	**	**
LM	12	0.55	ns	ns
MM	14	0.55	ns	ns
HM	16	0.55	ns	ns
LH	12	0.80	ns	ns
MH	14	0.80	ns	***
HH	16	0.80	ns	ns

*Note:* Significance: *α = 0.05, **0.05 < *α* < 0.01, ***0.01 < *α* <0.001; ns, not significant.

To assess whether the sensory differences highlighted by the TTs (Table 2) were driven by variations in VOC release induced by differences in matrix composition, the quantification of γ‐C9 and MLac volatilisation was carried out through HS‐SPME‐GC–MS in the headspace of the nine matrices. Histograms in Fig. 1(A), (B) depict the percentage of added γ‐C9 and MLac released from the nine matrices described in Table 1.

**Figure 1:**
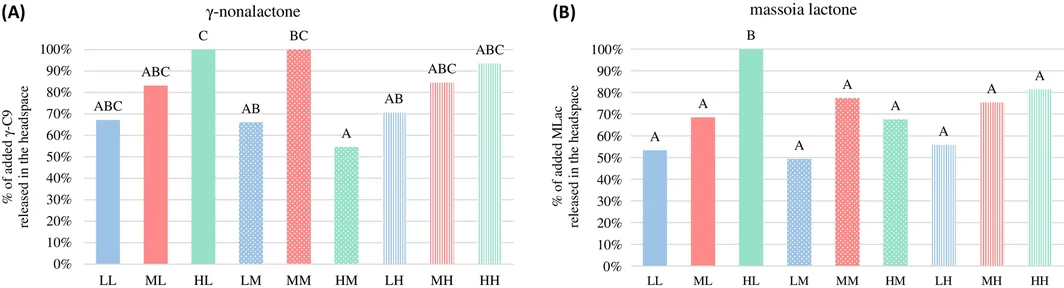
Percentage of added γ‐nonalactone (μg L^−1^) (A) and massoia lactone (μg L^−1^) (B) released in the headspace of the nine reconstituted wine matrices designed to simulate different wine profiles under varying climate change scenarios. Headspace concentrations were quantified by HS‐SPME‐GC–MS using external calibration curves and are represented as percentage of the spiked amount (155 μg L^−1^ for γ‐nonalactone and 26.8 μg L^−1^ for massoia lactone). All data are presented as the means of three replicates. Statistically significant differences among samples are indicated by different letters (ANOVA, *P* < 0.05; Tukey's HSD test). Each matrix was assigned a two‐letter code, in which the first letter refers to ethanol and the second to the pH/TA ratio, as follows: LL (12%, 0.4), ML (14%, 0.4), HL (16%, 0.4), LM (12%, 0.55), MM (14%, 0.55), HM (16%, 0.55), LH (12%, 0.8), MH (14%, 0.8), and HH (16%, 0.8). [Correction added on 13 July 2026, after first online publication: Figure 1 has been updated.]

The behaviour observed at low and high pH/TA ratios, corresponding to conditions of high and low ionic strength, suggests that volatile release is primarily driven by increasing ethanol concentration and, consequently, by ethanol‐related interfacial phenomena. As a tentative mechanistic explanation, these interfacial effects may be driven by the Marangoni effect. This interpretation is supported by the fact that the most pronounced and statistically significant increase in headspace concentration for both compounds was observed in the HL matrix (16% *v/v* ethanol; pH/TA 3.2/8), where high ethanol content co‐occurs with the highest ionic strength. Under this condition, the combined action of ethanol‐induced interfacial effects and salting‐out phenomena likely promotes enhanced mass transfer of volatiles from the liquid phase to the headspace. This mechanism appears more pronounced for γ‐C9, which is characterised by a lower boiling point (243 °C) and higher polarity (log*P*_o/w_ = 1.942) compared to MLac (boiling point 286 °C; log*P*_o/w_ = 2.540). These physicochemical properties make γ‐C9 more responsive to ethanol‐driven reductions in surface tension and polarity of the hydroalcoholic matrix, as well as to changes in volatilisation dynamics. A tendency towards increased γ‐C9 release was already observable – although not statistically significant – at intermediate ethanol levels (14% *v/v*), likely reflecting its higher concentration and greater sensitivity to ethanol‐mediated effects compared to MLac.

At intermediate ionic strength conditions, the observed behaviour is less straightforward to interpret and deviates from a simple monotonic trend. A tentative hypothesis is that, from LM to MM matrices, increasing ethanol still favours volatile release, with Marangoni‐driven interfacial effects likely remaining dominant. However, a further increase in ethanol content appears to counteract this tendency, possibly due to enhanced solubilisation of the compounds within the hydroalcoholic matrix, which may reduce the propensity of the volatiles to partition into the headspace, partially offsetting ethanol‐driven volatilisation effects.

Sensory results on the HL matrix are supported by GC chemical analyses on γ‐C9 and MLac release. Nonetheless, other significant sensory differences highlighted by the TT (Table 2) were not reflected by corresponding significant changes in headspace concentrations of γ‐C9 and MLac (Fig. 1(A),(B)), indicating that some sensory effects may not result directly from changes in the release of γ‐C9 and MLac, but instead from perceptual interactions and cognitive processes. It has already been shown that variations in the matrix composition can alter the qualitative odour profile and the overall sensory character of red wine even without significant modifications in the volatile composition or overall perceived odour intensity.^12^ Such changes cannot be fully explained by matrix‐driven modulation of VOCs release alone, as aroma release does not always account for sensory perception due to physiological mechanisms operating at both peripheral and central levels in the brain.^64^ In this regard, ethanol is a key component, and the sensory quality of wine seems to vary with its content following non‐linear patterns with distinct maxima and minima that are significant even with little ethanol increments. In red wine, these dynamics appear to induce perceptible discontinuities in the sensory profile at ethanol concentrations around 13–13.5% and 14.5–15%, with significant shifts observed not only in taste and tactile sensations but also in odour perception. Such transitions strongly support the role of ethanol as a key driver of both low‐ and high‐level cognitive processing during wine evaluation.^42^

Taken together, these results indicate that compositional changes linked to CC, particularly increased ethanol and a modified acidity profile, can substantially reshape the orthonasal aroma of wine. The detectability of γ‐C9 and MLac was not determined solely by ethanol or acidity, but by their combined action mediated by the matrix, confirming an interactive role of matrix composition in modulating both aroma release and perception. In particular, in red wine matrices with an ‘optimal’ pH/TA ratio, γ‐C9 became progressively more perceptible with increasing ethanol, while MLac was detected only at the maximum ethanol concentration (16% *v/v*), suggesting that the orthonasal sensory expression of both lactones is modulated by ethanol–acidity interactions within the matrix rather than by the only presence of the odourants. These findings are particularly relevant in the context of CC, where corrective practices such as additive acidification are increasingly applied to counteract acidic imbalances. According to our findings, it seems that in high‐alcohol matrices, acidity adjustments may unintentionally intensify the perception of climate‐related aroma markers such as γ‐C9 and MLac, potentially altering the aroma equilibrium.

### Influence of the matrix composition on flavour perception

The second objective of this study was to assess whether the modifications in wine matrix composition could have an influence on flavour perception in wines containing γ‐C9 and MLac, with particular emphasis on retronasal aroma perception and taste. To this end, a RATA was performed on the nine reconstituted wine matrices.

As a first step, retronasal aroma perception was investigated. Figure 2 reports the PCA illustrating the relationships between quantitative sensory data related to retronasal aroma attributes and mouthfeel (body) (variables) and the different wine matrices (observations). The first two principal components explained 82.49% of the total variance (F1: 59.46%; F2: 23.03%) and clearly revealed a composition‐driven sensory separation among samples. Along the first component (F1), matrices were divided into two main groups. Samples positioned on the positive semi‐axis (mainly HH and MH) were associated with higher body perception, chemical/ethereal nuances, and, to a lesser extent, sweet‐related notes and off‐odours. In contrast, matrices located on the negative semi‐axis (LL, LM, ML) were correlated with citrus and tropical fruit descriptors. The two matrices with opposed composition (LH and HL) were contraposed along the second component (F2).

**Figure 2:**
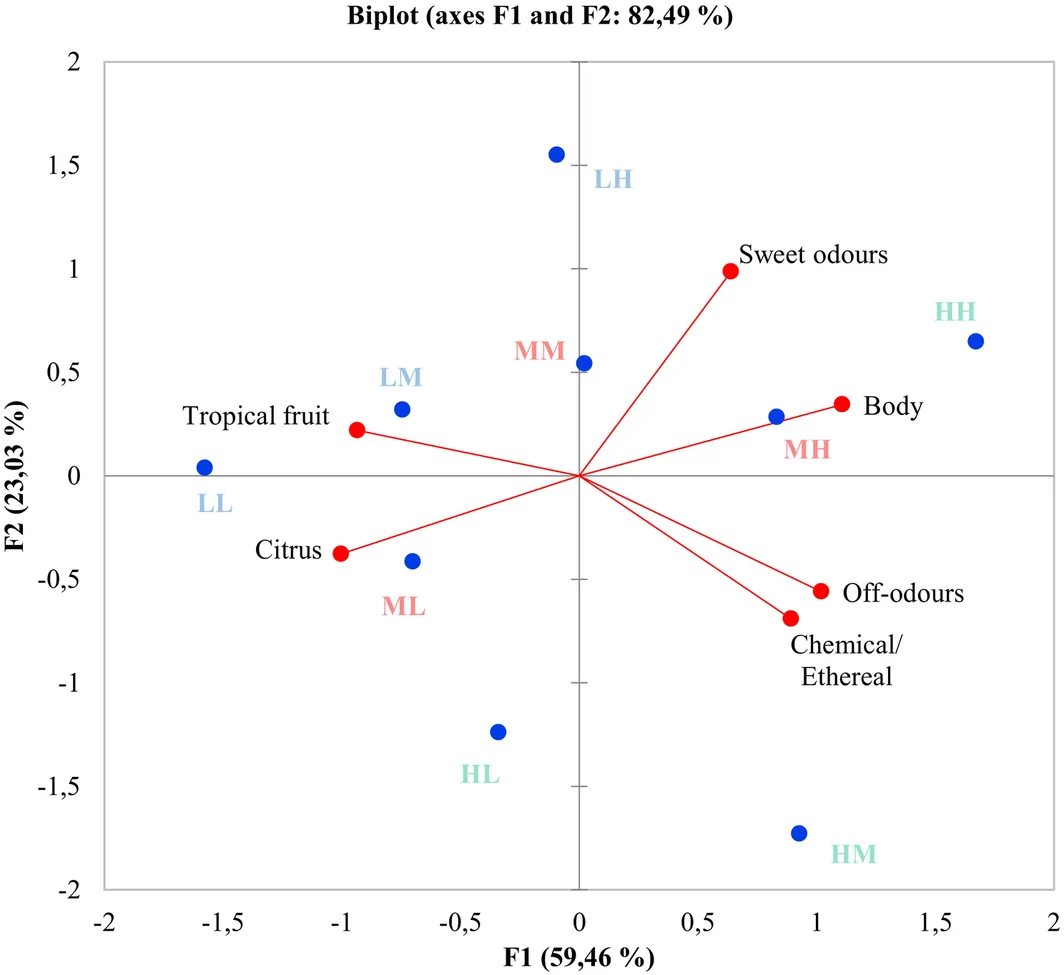
Principal component analysis (PCA) biplot based on sensory data (two repetitions) of aroma and mouthfeel (body) attributes (variables) for the nine reconstituted wine matrices (observations). PCA was performed using the average sensory scores for tropical fruit, citrus, sweet odours, off‐odours, chemical/ethereal, and body descriptors. Only descriptors with statistically significant contributions were included in the computation. Each matrix was assigned a two‐letter code, in which the first letter refers to ethanol and the second to the pH/TA ratio, as follows: LL (12%, 0.4), ML (14%, 0.4), HL (16%, 0.4), LM (12%, 0.55), MM (14%, 0.55), HM (16%, 0.55), LH (12%, 0.8), MH (14%, 0.8), and HH (16%, 0.8).

This distribution indicates that, at low and medium ethanol levels, increasing the pH/TA ratio (i.e., decreasing total acidity and increasing pH) progressively shifted the retronasal aromatic profile of matrices supplemented with a fixed amount of the two lactones from fresh citrus and tropical fruit notes (LL, ML) towards sweeter aromas and a greater perception of body (LH, MH).

When the ethyl alcohol content was high, the citrus and tropical fruit notes did not emerge (HL) and, moving from a low pH/TA ratio to medium (HM) and high (HH), it is hypothesised that ethanol progressively shifted from masking fruit notes to imparting an ethereal impression at the retronasal aromatic level, and ultimately body and sweet aromatic notes. This suggests that, under these conditions of high ethanol concentrations, its sensory role dominates and is modulated by the pH/TA ratio. Looking at the chemical/ethereal descriptor, matrices with low and medium ethanol content (12 and 14% *v/v*) were not correlated with this attribute, whereas high ethanol matrices (16% *v/v*), especially HM, showed a clear association. This suggests that up to 14% *v/v* ethanol, the aromatic character imparted by γ‐C9 and MLac (coconut, peach, and dried‐fruit notes) may contribute to masking ethanol‐related sensations. At 16% *v/v*, however, ethanol‐related cues became perceptually dominant, particularly under intermediate acidity conditions (medium pH/TA ratio). Similar masking effects have been reported in the literature, where fruity or tropical odourants attenuated ethanol‐associated notes,^40^ and combinations of woody and fruity compounds (e.g., whiskey lactone and isoamyl acetate) reduced the perception of ethereal or alcoholic character at high ethanol levels.^65^ In the present study, γ‐C9 and MLac appear to exert a comparable masking effect, but only within a limited compositional window (≤ 14% *v/v*), beyond which ethanol‐driven sensations prevail. The positive correlation between high ethanol matrices and increased body perception is consistent with sensory evidence indicating that ethanol contributes to mouthfeel by enhancing body, viscosity, and other sensations such as warmth and bitterness.^66^, ^67^ Even when viscosity‐related compounds (e.g., glycerol and polysaccharides) are held constant, higher ethanol levels are frequently associated with fuller‐bodied and more structured perceptions. Moreover, interactive effects between ethanol and acidity have been reported, with wines containing approximately 15% *v/v* ethanol at pH 3.4–3.6 described as exhibiting enhanced heat, viscosity, and velvety mouthfeel.^68^ In parallel, reduced acidity – particularly when combined with high ethanol – may favour the perception of sweet and ethereal notes, contributing to a progressive loss of aromatic freshness.^19^, ^42^, ^69^

Moreover, although the same concentration of lactones was added to all nine matrices, their role as potential modulators of oral and mouthfeel sensations cannot be excluded. A recent study suggested that medium‐chain lactones may contribute to mouthfeel perception independently of their odour activity. In particular, Kitajima *et al*.^70^ reported that several γ‐ and δ‐lactones with C9–C11 carbon chains, including γ‐C9, can act as taste modulators by activating the calcium‐sensing receptor (CaSR) in the oral cavity, thereby enhancing kokumi‐related sensations such as mouthfulness, thickness, and continuity (all linked to body), even under olfactory deprivation.^70^ Our findings seem in line with the observation that lactones may contribute to oral and mouthfeel perception beyond their aromatic role. In our experimental HH matrix, the two lactones could play a role in body perception, together with ethanol and the modulation of acidity. This hypothesis should be tested through specific experimental trials.

From a mechanistic point of view, these findings support the hypothesis that wine matrix composition modulates retronasal aroma perception not only through changes in volatility, but also via chemical–physical release dynamics and possible perceptual interactions. In agreement with this interpretation, a recent study on in‐mouth aroma release of six fruity esters (e.g., ethyl butanoate, isoamyl acetate, ethyl pentanoate, ethyl hexanoate, ethyl octanoate, and ethyl decanoate) in rosé wines suggested that increasing ethanol content enhanced the immediate release of more polar compounds while reducing the release of less polar ones.^43^ Therefore, although γ‐C9 and MLac concentrations were kept constant in the present study, matrix‐driven differences in partitioning, release kinetics, and perceptual integration (e.g., masking and dominance relationships among odour qualities) can account for the observed qualitative differences in retronasal aroma profiles.

The influence of ethanol and acidity on gustatory balance – specifically sweetness, sourness, bitterness, and sapidity – was further evaluated using a RATA test conducted under nose‐clip conditions to exclude retronasal olfaction and focus exclusively on taste perception.

Figure 3 presents the PCA of quantitative gustatory data, accounting for 93.94% of the total variance on the first two components (F1: 73.98%; F2: 19.96%). Along F1, matrices associated with sweet and bitter tastes (positive semi‐axis) were clearly separated from those correlated with sourness and sapidity (negative semi‐axis). As expected, increasing pH/TA ratios – reflecting decreasing total acidity and increasing pH – were associated with reduced sourness perception.^71^ Samples characterised by higher pH/TA ratios (MH, HH, as well as MM and HM) were projected on the positive side of F1, where they showed stronger correlations with sweetness and bitterness and a clear opposition to sourness. In addition, increasing ethanol content was generally associated with further attenuation of sourness perception. At the highest ethanol level (16% *v/v*) combined with high pH/TA ratio (low acidity) (HH), wines exhibited a marked reduction in perceived sourness and an increase in bitterness.

**Figure 3:**
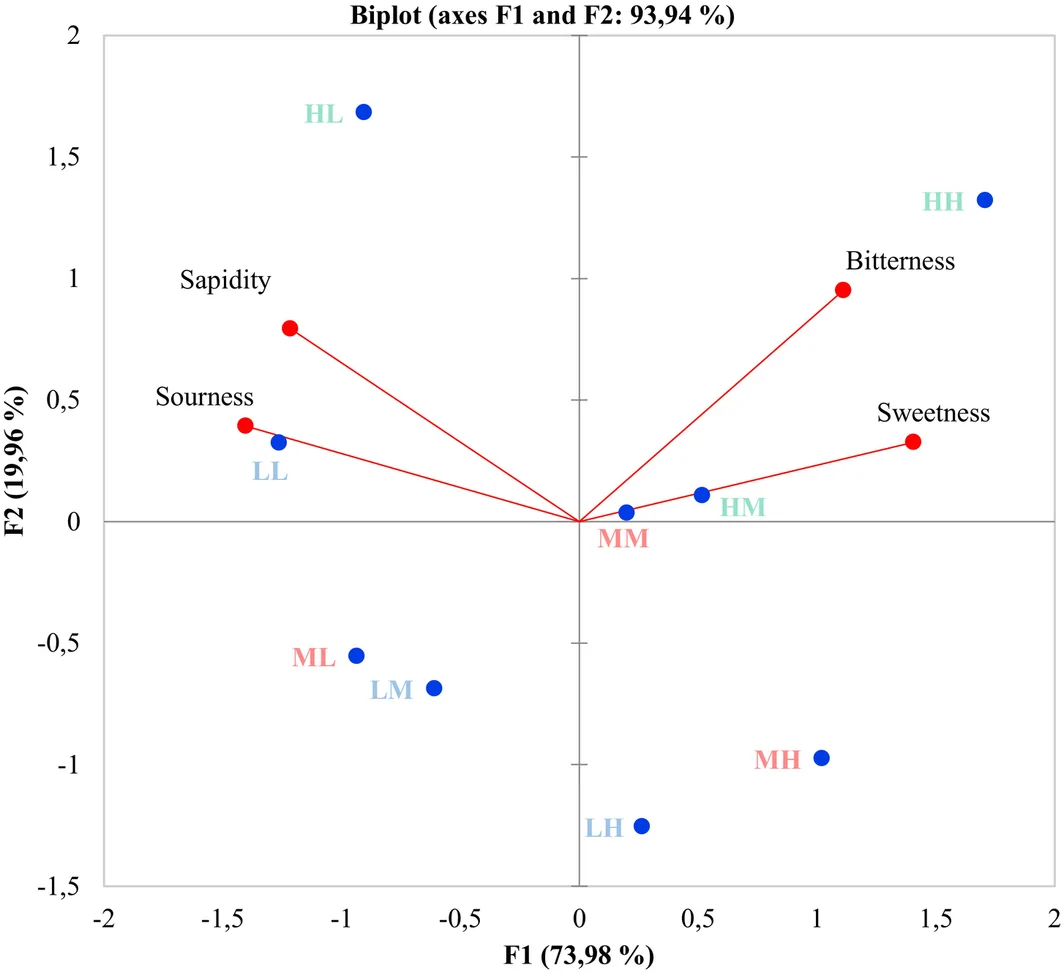
Principal component analysis (PCA) biplot based on sensory data (two repetitions) of in‐mouth attributes (variables) for the nine reconstituted wine matrices (observations). PCA was performed using the average sensory scores for saltiness, sourness, bitterness, and sweetness. Only descriptors with statistically significant model contributions were included in the computation. Sensory evaluation sessions for in‐mouth characteristics were conducted with participants wearing nose‐clips. Each matrix was assigned a two‐letter code, in which the first letter refers to ethanol and the second to the pH/TA ratio, as follows: LL (12%, 0.4), ML (14%, 0.4), HL (16%, 0.4), LM (12%, 0.55), MM (14%, 0.55), HM (16%, 0.55), LH (12%, 0.8), MH (14%, 0.8), and HH (16%, 0.8).

These results indicate that ethanol and acidity act interactively on gustatory perception. Increasing ethanol concentration contributes to intensified bitterness (Fig. 3) and body perception (Fig. 2), while higher pH values attenuate sourness (Fig. 2), resulting in an overall shift in taste balance towards less fresh and more bitter profiles.^42^, ^44^, ^72^ These results, together with those on retronasal aromatic perception (Fig. 2), indicate that the loss of freshness of HH, and even HM and MH involves both olfactory and in‐mouth sensations.

Changes can be partly attributed to cross‐modal taste–aroma interactions, whereby reduced acidity and increased ethanol enhance viscosity and sweetness perception, amplifying bitterness and suppressing sourness.^68^ The observed patterns are consistent with previous studies reporting that elevated ethanol levels and higher pH values promote bitterness perception while reducing sourness.^73^, ^74^

From a mechanistic point of view, it has been reported that ethanol can induce increases in viscosity and oral coating, which can alter the temporal and textural perception of taste stimuli, as well as modulate taste receptor sensitivity and the partitioning of taste and aroma compounds within the wine matrix.^44^, ^68^ The threshold effect observed in the present study further suggests that moderate compositional variations may not be sufficient to elicit perceptible sensory changes, whereas larger shifts in ethanol and acidity – such as those simulated here – can produce measurable alterations in gustatory balance.^75^ Sensory effects do not vary linearly with compositional changes, and the extent of the change may differ depending on the compositional range.

Under the hypothetical climate‐related conditions simulated here, such compositional combinations may lead to a loss of freshness and a disruption of gustatory balance, potentially affecting perceived quality and consumer appreciation. Therefore, from an applied enological perspective, these findings indicate that correcting a single compositional parameter as a punctual intervention (e.g., acidity or ethanol) may not be sufficient to acheive an overall sensory quality. Instead, a holistic approach is required, accounting for the interactions among ethanol, acidity/pH, other key wine matrix components, and volatile compounds. Such an integrated approach is essential to preserve the aromatic freshness and overall sensory harmony of red wines which, due to compositional imbalances linked to CC, are showing premature losses of freshness with sensory homologation.

## CONCLUSIONS

This study demonstrates that rising temperature‐driven modifications of red wine matrix composition related to CC, such as elevated levels of volatile lactones (γ‐C9 and MLac), increasing ethanol and converging pH and TA with its enological correction, significantly influence both the release and the flavour perception of red wines. The combined chemical and sensory approach supported our working hypothesis, highlighting that the sensory impact of γ‐C9 and MLac is not determined solely by their concentration, but largely by matrix‐dependent interactions between ethanol and acidity.

From an orthonasal perspective, variations in ethanol and pH/TA modulated the headspace availability of γ‐C9 and MLac, with the highest release observed under conditions of high ethanol and low pH/TA. In these matrices, sensory discrimination of both lactones was primarily driven by enhanced volatilisation, confirming that matrix composition governs aroma expression through physicochemical mechanisms such as changes in partitioning and mass transfer. However, several perceptible differences were not fully explained by headspace concentrations alone, suggesting that matrix‐driven perceptual and cognitive mechanisms may also contribute to odour expression beyond simple release phenomena.

At the flavour level, ethanol and acidity acted as interactive sensory modulators. Increasing ethanol combined with decreasing acidity shifted retronasal perception from fresh citrus and tropical notes towards sweeter odours and ethereal notes, while also enhancing body perception. In parallel, gustatory balance was affected, with matrices characterised by high ethanol and high pH/TA perceived as less sour and more bitter, consistent with cross‐modal interactions, in which ethanol‐related mouthfeel and sweetness suppress acidity and favour bitterness expression. These results, together with those on retronasal aromatic perception, indicate that high ethanol combined with converging pH and TA, which occur in the actual climatic context, contribute to a loss of freshness involving both retronasal olfactory and taste sensations.

From an enological perspective, this has practical implications: the frequent practice of acidification used to correct pH/TA convergence may, when applied in wines with high ethanol and elevated lactone levels, further increase the sensory impact of γ‐C9 and MLac rather than simply restoring freshness. In this sense, although the present work was developed in the context of CC, it may also be of interest for improving the sensory quality of low‐alcohol and dealcoholised wines, where changes in ethanol and acidity are known to affect aroma release and perception. This work, which is among the first to address CC effects through a matrix–aroma interaction framework, nevertheless has some limitations. The experiments were conducted on deodourised wines and did not consider other key compositional fractions (e.g., the polyphenolic matrix). In addition, γ‐C9 and MLac were added at relatively high concentrations, selected to reflect upper levels reported in the literature, which may have amplified sensory effects and limit direct extrapolation to typical commercial wines. Future studies should therefore validate the findings of this pilot study.

## CONFLICT OF INTEREST

The authors declare that they have no known competing financial interests or personal relationships that could have appeared to influence the work reported in this article.

## Data Availability

The data that support the findings of this study are available from the corresponding author upon reasonable request.
